# Mafic glass compositions: a record of magma storage conditions, mixing and ascent

**DOI:** 10.1098/rsta.2018.0004

**Published:** 2019-01-07

**Authors:** Katharine V. Cashman, Marie Edmonds

**Affiliations:** School of Earth Sciences, University of Bristol, Bristol BS8 1RJ, UK

**Keywords:** melt, mixing, degassing, gas fluxing, trans-crustal, magma reservoir

## Abstract

The trans-crustal magma system paradigm is forcing us to re-think processes responsible for magma evolution and eruption. A key concept in petrology is the liquid line of descent (LLD), which relates a series of liquids derived from a single parent, and therefore tracks the inverse of the crystallization path. It is common practice to attribute multiple magma compositions, and/or multiple melt compositions (from melt inclusions and matrix glass), to a single LLD. However, growing evidence for rapid, and often syn-eruptive, assembly of multiple magma components (crystals and melts) from different parts of a magmatic system suggests that erupted magma and melt compositions will not necessarily represent a single LLD, but instead may reflect the multiple paths in pressure–temperature space. Here, we use examples from mafic magmatic systems in both ocean island and arc settings to illustrate the range of melt compositions present in erupted samples, and to explore how they are generated, and how they interact. We highlight processes that may be deduced from mafic melt compositions, including the mixing of heterogeneous primitive liquids from the mantle, pre-eruptive magma storage at a range of crustal and sub-Moho depths, and syn-eruptive mixing of melts generated from these storage regions. The relative dominance of these signatures in the glasses depends largely on the water content of the melts. We conclude that preserved melt compositions provide information that is complementary to that recorded by the volatile contents of crystal-hosted melt inclusions and coexisting mineral compositions, which together can be used to address questions about both the pre- and syn-eruptive state of volcanic systems.

This article is part of the Theo Murphy meeting issue ‘Magma reservoir architecture and dynamics’.

## 1. Introduction

Recent reviews of magmatic systems (e.g. [1–6]) emphasize their spatial extent, dynamic nature and rheological complexity. This view of magmatic systems has evolved over the past decades, driven by the accumulation of geophysical, petrological and geochemical evidence that active magmatic systems are vertically and/or laterally extensive and are dominated by crystal mush, with only transient accumulations of crystal-poor melt. Recognition of the transience and the mobility of the melt, combined with the high inferred crystallinity of most sub-volcanic systems, is prompting the community to re-think processes responsible for both magma evolution and volcanic eruptions, particularly related to redistribution of melt and crystals within magma storage regions.

Petrology has contributed to the paradigm of trans-crustal magmatic systems (TCMS) by improving constraints on the pressure (*P*) and temperature (*T*) of magma storage using experimentally derived phase relations and thermobarometric models [7–10]. At the same time, direct measurements of dissolved volatile components in crystal-hosted melt inclusions and quenched matrix glass can be used to track the movement of volatile phases through the system [11–13]. Thermodynamic models used to reconstruct crystallization paths, including MELTS [14] and Petrolog [15], and degassing paths, e.g. VolatileCalc [16] and D-Compress [17], extend experimental constraints and allow construction of melt evolution paths for changing conditions of pressure, temperature and composition. New microanalytical techniques permit detailed studies of the entrained crystal populations. Diffusion profiles measured across internal boundaries within crystals are now routinely used to estimate residence times of crystals within the transporting melt and, by extension, time scales of pre-eruptive magma accumulation (e.g. [18–20]). In combination, these approaches have shown that any single batch of erupted magma may combine crystals and melt from a range of *P–T* conditions within a given magmatic system [21–23].

**Figure 1.**
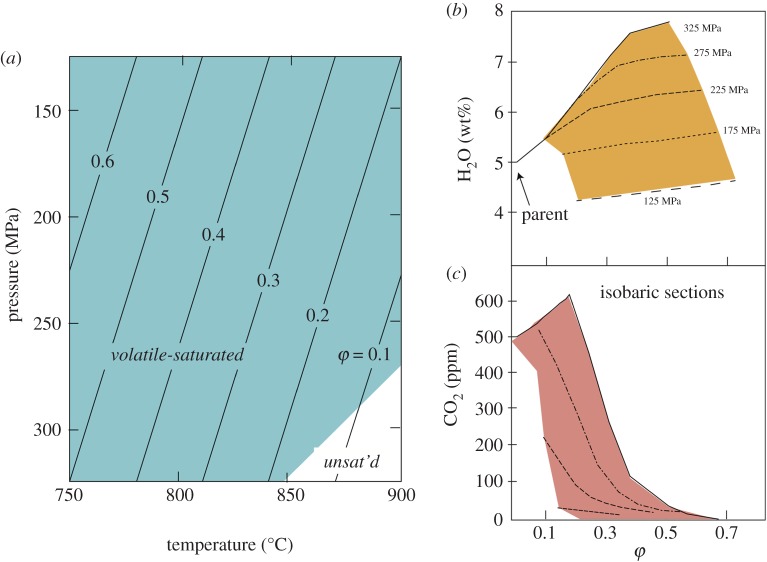
Evolution of a model intermediate-composition magma chamber. (*a*) Model magma chamber set-up in *P–T* space; blue region indicates volatile saturation, contours are lines of equal crystal volume fraction (*φ*). Isobaric sections show variations in (*b*) H_2_O and crystallinity and (*c*) CO_2_ and crystallinity. Reproduced with permission from [24].

A schematic magma storage system is shown in figure 1*a*, which spans a range of pressure (*P*), temperature (*T*) and crystallinity (*φ*). As a result, the hypothetical resident magma can be vapour-undersaturated (at high *P* and *T*) or vapour-saturated, and ranges from pure melt (also at high *P* and *T*) to ‘non-eruptible’ (*φ *> 0.6) crystal mush [24]. Such a model system has the potential to trap melt inclusions (MIs) with a wide range of H_2_O and CO_2_; this forms the basis of using crystal-hosted MIs to infer conditions of magma storage (figure 1*b,c*) [16]. The same system will have a large range of melt compositions that also reflect the *P–T*–*φ* conditions of magma storage; it is this melt compositional range that we explore in this paper. Melt may be trapped as inclusions within crystals, preserved as a matrix phase of the transporting magma and is also commonly preserved as a matrix phase within glomerocrysts and cumulate nodules, or surrounding antecrysts (antemelt) [25,26]. Glass compositions can provide a detailed record of the heterogeneity of ‘primary’ melts [27], magma recharge and mixing [28,29], ascent rate [30] and syn-eruptive mixing [31,32], particularly in hydrous mafic magmas that crystallize rapidly during decompression. Here we explore glass compositions of both relatively dry ocean island tholeiites and hydrous arc basalts to examine the types of processes they record, and discuss their utility for understanding magmatic storage and transport processes in a range of tectonic settings. We focus on basaltic magmas because they occur in most tectonic settings, represent the less evolved input to magmatic systems, and vary widely in H_2_O content, and therefore in conditions of crystallization.

## 2. Exploring melt evolution over *P–T* space

### (a) Model mafic magmatic systems

**Figure 2.**
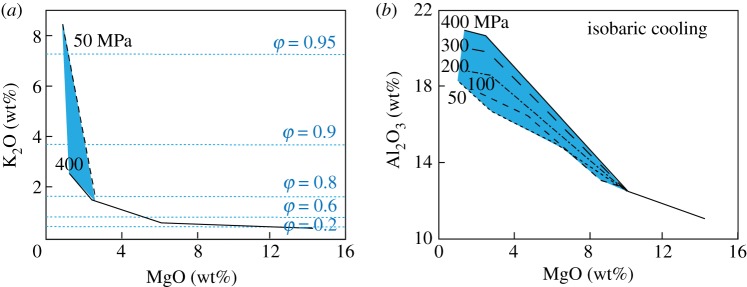
Evolution of melt composition during isobaric cooling for a H_2_O-poor tholeiite. (*a*) K_2_O versus MgO, with crystallinity (*φ*) and pressure marked. (*b*) MgO versus Al_2_O_3_, with pressures marked. Melt compositions from rhyolite-MELTS [14]. (Online version in colour.)

**Figure 3.**
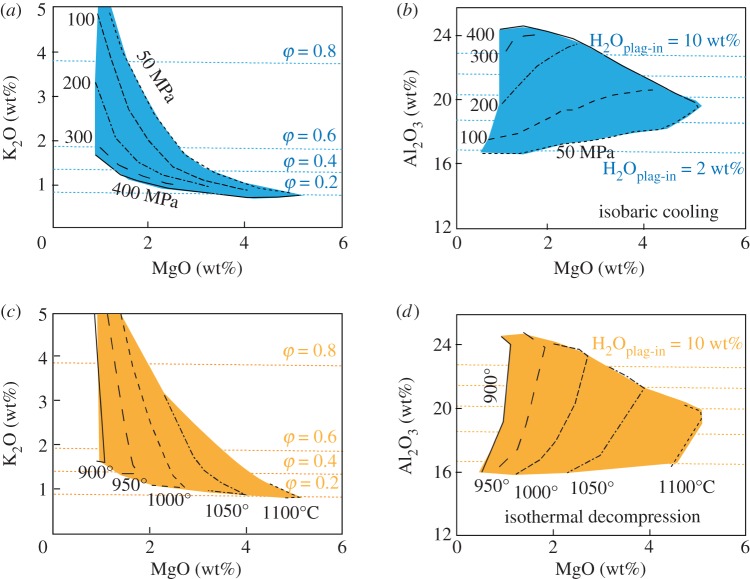
Evolution of melt composition during IBC (*a,b*) and ITD (*c,d*) for an H_2_O-rich arc basalt. (*a,c*) K_2_O versus MgO, with crystallinity (*φ*) and pressure marked. (*b,d*) MgO versus Al_2_O_3_, with pressures marked. The starting composition was an estimate of the bulk composition for the 1974 Fuego eruption (from [37]) with 4.5 wt% H_2_O and *f*O_2_ of NNO. Compositional paths determined from rhyolite-MELTS [14] run from 400 to 50 MPa and 1100 to 900°C. The compositional space covers most of the observed range of melt compositions at Fuego, and illustrates the compositional spectrum that may arise when magmas are tapped during a single eruption from a range of *P–T* space within a complex magmatic system. (Online version in colour.)

To develop a framework for examining melt compositions, we first develop simple model systems similar to that shown schematically in figure 1. As analogue systems we have chosen Kīlauea volcano, which erupts tholeiitic basalt with approximately 0.7 wt% H_2_O [33,34], and Fuego volcano, Guatemala, a frequently active and well-studied volcano with a hydrous high-alumina basalt composition [35–37]. For the Kīlauea model, we use MELTS to model equilibrium crystallization at a range of pressures, using a starting composition equivalent to the most primitive post-entrapment crystallization (PEC) corrected melt inclusion from [34] which is similar in composition to the primitive glasses from the Puna Ridge [38], and an *f*O_2_ of FMQ (fayalite–magnetite–quartz) [39]. For the Fuego model, we use MELTS and a starting composition appropriate for the bulk Fuego 1974 magma (from [37]). To reduce the number of variables, we use an initial H_2_O content of 4.5 wt% (from [40]) and assume that *f*O_2_ is buffered at NNO (nickel–nickel oxide) (e.g. [41]). For simplicity, we assume equilibrium crystallization paths; fractional crystallization would further enhance the modelled trends. The model space ranges from 1100 to 900°C and from 400 to 50 MPa. We examine end member paths of isobaric cooling (IBC; figures 2 and 3*a,b*) and isothermal decompression (ITD; figure 3*c,d*) as illustrated by plots of MgO (to track crystallization of mafic phases) versus K_2_O to track total crystallinity (assuming that it behaves incompatibly) and Al_2_O_3_ to indicate the onset of feldspar crystallization, which is particularly sensitive to $P_{H_{2}O}$.

The Kīlauea model (figure 2) shows that in H_2_O-poor ocean island tholeiites, the major element composition of the melt is not sensitive to the pressure of crystallization for much of the cooling history; for this reason, IBC trends form a single liquid line of descent (LLD) until the melt MgO content reaches approximately 7–9 wt%, when plagioclase and pyroxene appear on the liquidus. At this point, the influence of plagioclase crystallization on Al_2_O_3_ increases as the pressure of crystallization decreases, leading to 2–4 wt% differences in Al_2_O_3_ content at a MgO content of 6 wt%. This model system shows that, in low-H_2_O basaltic magmas, the melt compositions are relatively insensitive to d*P*/d*T* paths and instead are much more likely to preserve heterogeneity in major and trace elements (and in isotopic composition) that may be linked back to a variable mantle source composition and degree of melting [42–45].

For more hydrous melts in arc settings, in contrast, the evolution of the melt phase is strongly controlled by pressure, as illustrated by the Fuego model (figure 3). Compositional trends produced by IBC at high pressure are controlled by crystallization of mafic phases, with accompanying strong decreases in MgO and associated increases in Al_2_O_3_, with only minor increases in K_2_O (total crystallinity; figure 3*a,b*). The MgO content at which plagioclase saturation occurs varies strongly with the crystallization pressure, and is marked by the maximum Al_2_O_3_ content of the melt (Al_2_O_3_^max^) [46]. Al_2_O_3_ then decreases with decreasing pressure as plagioclase crystallizes. Associated increases in bulk crystallinity are indicated by a rapid steepening of the MgO–K_2_O curves. During early phases of IBC crystallization, these feldspar-driven MgO–K_2_O trends are more pronounced at lower pressures because plagioclase crystallization starts earlier in the crystallization sequence, as illustrated by the changing location of Al_2_O_3_^max^. The reverse is true of MgO–K_2_O curves in the later stages of crystallization, which are steepest at the highest pressure and represent rapid feldspar crystallization once H_2_O saturation is reached. Compositional trends produced by ITD, in contrast, start with decompression-driven melting of clinopyroxene, which causes an initial increase in MgO. This is followed by rapid decreases in Al_2_O_3_ and increases in K_2_O that record extensive decompression-driven plagioclase crystallization (figure 3*c,d*). Both curves are steepest at the lowest temperatures. Although not shown, combined decompression and cooling produce melt evolution paths that traverse the same compositional space covered by the end member cases shown in figure 3. Taken together, these model curves provide an heuristic framework for examining the melt evolution paths preserved in the eruptive products of mafic volcanoes in ocean island and arc settings.

### (b) Experimental constraints on melt compositional paths

**Figure 4.**
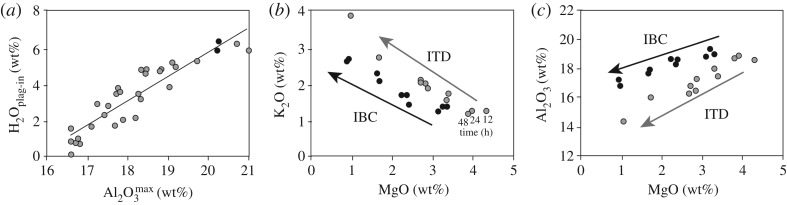
Experimental constraints. (*a*) Development of an Al_2_O_3_ hygrometer. Experimental data from [46] at 200 MPa with best-fit line (equation provided in text); additional data points from higher-pressure experiments of [51]. Standard error is 0.76 wt% H_2_O. (*b,c*) Dynamic experiments on H_2_O-saturated basaltic andesite starting at 1025°C and 150 MPa and with cooling to 925°C or decompressing to 10 MPa. End member effective supercooling (Δ*T*_eff_) values for feldspar are labelled. Data from [52].

Changes in melt composition related to cooling and changes in *P*_H_2_O_ can be calibrated experimentally. For example, variations in MgO caused by olivine crystallization (figure 2) form the basis of an experimentally calibrated glass geothermometer that was first developed for Kīlauea basalt compositions at 1 bar [47] and later extended to Mauna Loa [48] and Columbia River basalt [49] compositions. A generalized form of the MgO-glass geothermometer [50] is *T*(°C) = 26.3MgO^liq^ + 994.4°C, which has a standard error estimate of ±71°C, although the error can be reduced by adding compositional and pressure-dependent terms. Another example is calibration of a melt-based hygrometer [46], which is based on the relation between H_2_O saturation and plagioclase crystallization, as illustrated in figure 3*b*. The calibrated relationship between the H_2_O content of basaltic melts at plagioclase saturation (H_2_O_plag-in_) and Al_2_O_3_^max^ is H_2_O_plag-in_ = 1.34Al_2_O_3_^max^ − 21.05, as shown in figure 4*a*. Although the original calibration points were based on moderately low-pressure experiments (100–200 MPa, [46]), data from additional experiments at mid- to lower-crustal pressures [51] suggest that the calibration can also be extended to higher pressures. Together, these examples illustrate the potential of melt compositions to record both pre- and syn-eruptive $P_{H_{2}O}-T$ histories.

Experimental data can also be used to compare IBC and ITD crystallization paths, as illustrated by the experiments of [52] on a hydrous basaltic andesite. Here, a melt equilibrated under H_2_O-saturated conditions at 150 MPa and 1025°C was subsequently (a) cooled to temperatures of 995, 965, 935, 915 and 892°C at a constant pressure of 150 MPa (IBC) and (b) decompressed to 100, 65, 42, 21 and 10 MPa at a constant temperature of 1025°C (ITD). The initial condition of H_2_O saturation means that we capture only part of the composition space spanned in figure 3, but the crystallization paths are directly comparable in that experimental conditions were chosen to achieve the same effective supersaturations for plagioclase crystallization (the target of these kinetic experiments), and were either cooled or decompressed over the same time intervals. The resulting melt compositions vary with the *P–T* path, as illustrated by plots of Al_2_O_3_, K_2_O and MgO (figure 4*b,c*). Specifically, the ITD experiments show a more rapid decrease in Al_2_O_3_ and a more rapid increase in K_2_O than equivalent IBC experiments, consistent with the expected enhanced crystallization of plagioclase relative to mafic phases during decompression. The kinetic effects of varying experimental time scales, in contrast, are small.

In summary, both the model and experimental data illustrate the range of composition space that can be experienced by variably hydrous mafic melts that follow different *P–T* paths in a single magmatic system. These data have important implications for interpreting the range of melt compositions preserved in erupted material. Here we explore this approach using several rich datasets from mafic eruptions.

## 3. Melt compositions in mafic ocean island volcanoes

Mafic magmas in ocean island settings typically contain less than 1 wt% H_2_O [33,53,54], which means that they are not very susceptible to decompression-driven crystallization, and indeed are often erupted near their liquidus. Erupted magmas are therefore characterized by a low phenocryst content and a glassy groundmass. Geobarometric studies that have compared the compositions of coexisting glass and pyroxene [7,10] have shown that eruptions of ocean island volcanoes are supplied by melts that have been stored, prior to eruption, over a wide range of pressures [7,55,56], perhaps extending past the petrologic Moho into the upper mantle for some settings. Beneath Mauna Kea, Hawai‘i, which is in its post-shield phase, fractionation of clinopyroxene occurs during magma storage in the uppermost mantle (800 MPa) to yield hawaiitic magmas [57]. Post-shield magmas from Haleakala volcano, on the island of Maui, Hawai‘i, show evidence for magma storage at pressures up to 950 MPa [10,58]. At many ocean island volcanoes, there is little evidence of long-lived, shallow ‘magma chambers’ [59]. It has been proposed that magma reservoirs beneath Hawai‘i, for example, may develop through a cycle, whereby the early, pre-shield alkalic phase of activity is characterized by multi-stage magma storage, eventually forming long-lived magma reservoirs as the magma supply rate increases [59]. During the tholeiitic stage of volcanism, the dominant zone of magma storage becomes shallower, within a few kilometres of the surface. During this phase, the shallow magma chamber may fill and drain repeatedly; rapid drainage may be associated with caldera collapse. During the post-shield phase, when magma supply wanes, only the deep reservoir survives. The geometry and depth of magma reservoirs beneath ocean islands are not well resolved, however, and we must typically rely on a combination of petrology and geophysics to unravel their form and extent. Importantly, samples of quenched ‘carrier liquid’, which represents the melt phase, provide valuable clues about the depth of storage, fractionation, degassing and mixing of magmas. Here, we examine what the major and trace element composition of the melt for ocean island basalts can tell us about mantle source, mixing and the magmatic plumbing system, before we contrast it, in §4, with the case of more hydrous, mafic arc magmas.

### (a) Kīlauea volcano, Hawai‘i

Kīlauea volcano, Hawai‘i, is in its shield-building phase, with large magma fluxes and frequent eruptions. A dominant summit reservoir exists at 2–4 km depth [60,61] and a shallower one at approximately 1 km [62], both of which are well constrained by geophysical data. The rift and summit zones of magma storage may extend vertically downwards, perhaps to the inward-dipping decollement at approximately 10 km depth [63] where the edifice sits on the top of the pre-volcano crust. Magma ascends sub-vertically from the zone of melting at depths of greater than 40 km [64]. P-wave tomography reveals high-velocity regions beneath the summit and rift zones inferred to represent dense cumulates, as well as low-velocity regions that may represent melt beneath the summit of Kīlauea [65,66]. The detailed structure of the plumbing system beneath the summit magma reservoir, however, is very poorly known, and topics for debate include the extent to which magmas may ‘bypass’ the summit reservoir, the depth of lateral transport in the rift zones, and the extent of rift zone storage [63].

**Figure 5.**
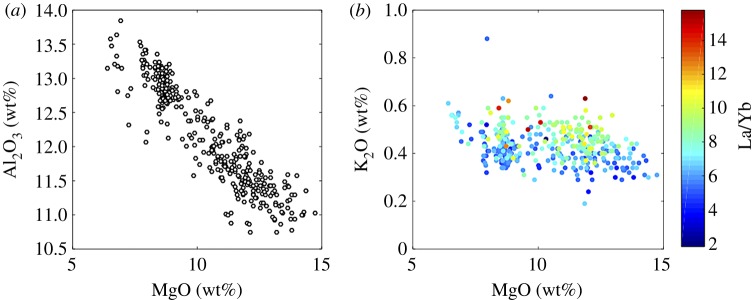
Olivine-hosted melt inclusion compositions in tephra erupted during 25 eruptions over the past 400 years at Kīlauea volcano, Hawai‘i, from [34]. (*a*) Al_2_O_3_ versus MgO and (*b*) K_2_O versus MgO colour-coded for La/Yb to show that some of the scatter in major element compositions is inherited from the heterogeneity of melts that came out of the mantle and mixed together in crustal magma reservoirs.

The magmas erupted at Kīlauea volcano are relatively simple petrographically, offering scant opportunity for barometric reconstructions of magma storage, as many melts erupt with only olivine on the liquidus. Olivine control dominates the LLD, with plagioclase and augite appearing on the liquidus at MgO contents of less than 7 wt%. Whole-rock compositions often exhibit signs of olivine accumulation [67,68]. Primary melts have approximately 16 wt% MgO [38] and fractionate approximately 30% of their mass as olivine to reach approximately 7 wt% MgO. Picritic glasses erupt exceedingly rarely, if at all, and are recorded only in dredged samples from the submarine portion of the Puna Ridge [38]. Figure 5 shows matrix glass and melt inclusion compositions for tephras erupted during 25 summit and upper East Rift Zone eruptions over the last 600 years of Kīlauea's activity [34]. Melt inclusions corrected for PEC reach almost 15 wt% MgO [34] while matrix glasses reach up to approximately 10 wt% MgO [69]. However, the relative insensitivity of basaltic glass composition to the pressure of crystallization (figure 2) means that the depth at which these olivines crystallize is very poorly constrained. The melt water content and *f*O_2_ also affect the timing of plagioclase and clinopyroxene fractionation with respect to temperature.

The simple petrographic relationships observed in Kīlauea magmas belie considerable complexity, however, related to mixing of heterogeneous melts and accumulation of antecrystic olivines. Together, these phases provide information about the thermal structure of the magma reservoir system (owing to the simple relationship between glass MgO content and temperature [47]; figure 2) and its physical arrangement. Matrix glass compositions overlap with melt inclusion compositions, and while their overall trends are related to olivine fractionation, they exhibit considerable variability in major (and trace) elements for a fixed MgO content. This variability in major element composition correlates with indices of mantle source variability, e.g. La/Yb (figure 5*b*) and Pb isotopic composition [43,70], which may reflect melt fraction ratios and/or lithology of the mantle source [43,71]. The heterogeneous melts present in the mantle beneath Kīlauea may also have different volatile contents. For example, it has been suggested that the ‘Loa’ trend may be associated with recycled dehydrated oceanic crust, and that the lower H_2_O and CO_2_ contents [34,72] may affect phase equilibria during fractionation in the crust. Thus, although they form a broad trend in compositional space, the data in figure 5 are clearly not described well by a single LLD; instead, it is likely that fractionation takes place from a range of primary melt compositions.

**Figure 6.**
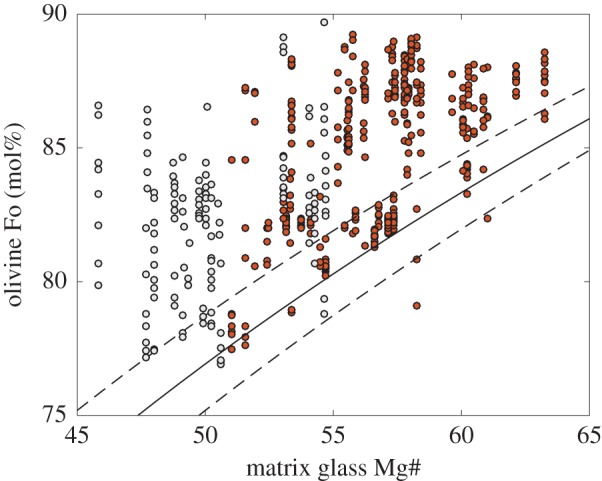
The antecrystic nature of olivines in ocean island basalts. Olivine core compositions plotted against the Mg# of the carrier liquid, for 25 eruptions of Kīlauea volcano over the past 400 years [34] (red) and morphologically young submarine cones from El Hierro (Canary islands) [75] (grey). The solid and dashed lines indicates crystal–melt equilibrium (where *K*_D_ = (*X*_FeO_/*X*_MgO_)_olivine_/(*X*_FeO_/*X*_MgO_)_melt_) of 0.3 ± 0.05 for olivine/liquid [76].

Matrix glass compositions also show considerable variability in MgO content and hence temperature (from 4.4 to 10.3 wt%; 1102–1221°C; figure 5) through space and time, even within individual eruptions [34,69]. This requires syn-eruptive mixing of liquids. In particular, extra-caldera eruptions of Kīlauea in 1959, 1971 and 1974 produced matrix glasses ranging from 6.5 to 9.0 wt% within the same deposit [69]. Explosive ash-rich summit eruptions show even wider ranges, producing tephra with 6.5–11.0 wt% (Keanakāko‘i; 1500–1800 AD) and less than or equal to 12.5 wt% (Kulanaokuaiki; *ca* 400–1000 AD). The widespread Pāhala ash (25–10 ka) includes rare shards with 13–14.5% MgO [73]. Intra-caldera glasses, in contrast, contain only 6.4–7.6 wt% MgO, reflecting compositions that are buffered at the peritectic, probably because of thermal constraints [74]. The extra-caldera eruptions also commonly produce tephra that contains more primitive olivines [34]. Comparison of the matrix glass composition of Kīlauea tephras with the compositions of the cores of their olivine cargoes (figure 6) reveals that the vast majority of the olivine crystals are not in equilibrium with their ‘carrier liquid’ [34,77], a feature earlier highlighted by a comparison between whole rocks and olivine compositions [68,78]. It is worth noting that olivines in the range Fo_86–87_ would be in equilibrium with melts of MgO content 10.5–11.0 wt%, which is only erupted at Kīlauea in ‘unmixed’ form during the most explosive eruptions (e.g. the Keanakāko‘i and Kulanaokuaiki eruptions). Evidence for magma mixing is pervasive in these olivine crystals, which show resorption and normal and reverse zoning [18,79]; additional evidence for mixing lies in the heterogeneity of melt compositions. The 1959 Kīlauea Iki eruption, for example, involved mixing of an invading high-temperature magma into a cooler, shallower stored magma [47,80–83].

These observations have prompted a model of a vertically zoned shallow magma reservoir beneath the summit, with more primitive melt (and olivines) being sourced from deeper in the system (greater than 2–3 km) and erupted only during (1) explosive eruptions from the summit, (2) extra-caldera eruptions during which magma may bypass the shallowest magma storage area [34] and/or mix with shallower stored melts [69] and (3) prolonged rift eruptions which may empty the summit reservoir. The lower and more homogeneous MgO contents of most summit eruptions, in contrast, are sourced from the well-mixed shallowest portion of the reservoir, which may change in composition over decades as the reservoir is recharged by melts from the mantle.

### (b) Volcanoes in the Canary Islands, Iceland and the Galapagos

The Canary Islands provide a contrasting view of ocean island magmatic systems and are associated with a lower magma flux. The resulting infrequent eruptions do not allow collation of detailed geodetic or volcano-seismicity datasets over long time scales, but instead require petrological techniques to reconstruct magma storage depths. Unlike Kīlauean magmas, Canarian magmas contain abundant clinopyroxene, reflecting the dominance of clinopyroxene over olivine fractionation at high pressures [84,85]. Barometry using clinopyroxene-melt compositions [7,9,10] for basalts erupted from the shield volcano Teno, on the island of Tenerife, suggests magma storage depths of 20–45 km [86]. Clinopyroxene-melt barometry for basalts on other Canary Islands also suggests the existence of sub-Moho magma reservoirs, which perhaps occur as a plexus of dykes and sills [75,87]. On La Palma, clinopyroxene-melt barometry indicates last equilibration pressures of about 400–700 MPa for magmas erupted from Cumbre Vieja volcano [88]; for El Hierro, 500–710 MPa [75]; and for magmas erupted on the Madeira archipelago, 400–1050 MPa [89,90]. Independent methods to estimate pressures produce similar results: the density of CO_2_-dominated fluid inclusions in olivines in xenoliths suggests magma storage depths of 650–950 MPa for El Hierro, 600–680 MPa for La Palma and 550–750 MPa for Lanzarote [56]. For many of the volcanic centres, the fluid inclusion systematics also highlight a shallower crustal magma staging area [56], similar to many volcanic systems in Iceland [7,91]. There is evidence for some shallow fractionation of magmas in the Canary Islands to form phonolites, and evolved plutonic nodules are erupted on, for example, Tenerife [92].

Like basalts from Hawai‘i, Canarian magmas often exhibit clear signs of mixing [86]. Here, however, mixing appears to take place in the deep, vertically disseminated, ephemeral magma reservoirs that straddle the (seismic) Moho. In El Hierro lavas [75], olivine cores are frequently not in equilibrium with their carrier liquid (figure 6). By comparing the least and most primitive olivine compositions, one might speculate that a crystal-rich, more evolved magma (and its crystal cargo) mixed with a picritic magma (carrying primitive olivines), or that invading melt disrupted a crystal-rich, compositionally zoned mush, picking up a range of olivine crystals shortly before erupting.

In summary, the structure of magmatic storage systems beneath ocean island volcanoes depends primarily on magma flux from the mantle, such that high fluxes allow development of shallow storage systems while low fluxes promote magma storage in lower-crustal sills. Early crystallized olivine that is physically removed from the melt can be re-incorporated by an intruding carrier liquid; the result is a mafic crystal cargo that is more primitive than the melt that transports it to the surface during eruption. Melt compositions provide evidence of extensive mixing of both deep and shallow magmas, but also of different melt batches from the mantle source. Matrix glass compositions erupted during different styles of eruption hint at a vertically zoned magma storage system beneath the summit of Kīlauea. Finally, variations in the spatial distribution of matrix glass compositions at Kīlauea demonstrate the utility of using a wide range of eruptive products to fully probe the subsurface magmatic system.

## 4. Melt compositions in mafic arc magmas

Mafic arc magmas are typically hydrous [93] and often high in alumina, which, when combined with the relatively low viscosity of the melts, makes them particularly susceptible to plagioclase-dominated decompression-driven crystallization. For this reason, typical erupted magmas have both a moderate to high phenocryst content and a moderately to highly crystalline groundmass. Geobarometry based on both mineral phases and crystal-hosted melt inclusions suggests that eruptions are typically fed from mid- to upper-crustal depths, rather than directly from the lower crust, although there are exceptions (e.g. [94]). Magmatic systems tend to be vertically extensive, with the deeper parts sampled only during the highest-energy eruptions [95,96]. Pre- and syn-eruptive entrainment of antecrysts is common [97,98], and isotopic data can record entrainment of antemelt [25]. Below we examine processes of recharge, storage and eruption through the lens of melt composition, which provides a complementary record to that of the volatile and crystal phases. Only a limited number of mafic arc volcanoes have extensive published melt compositional data. We have chosen to compare four such volcanoes: (1) Stromboli volcano, Italy, is best known for its eponymous frequent, shallow and low-level activity, which is punctuated by energetic paroxysms; (2) Etna volcano, Italy, is frequently active with recent eruptive styles that range from effusive to violent Strombolian; (3) Llaima volcano, Chile, is sporadically active, with the most recent eruptive phase in 2008–2009 characterized by both violent Strombolian and Strombolian eruptions as well as effusive activity; and (4) Fuego, Guatemala, is frequently active with eruptive styles that range from Strombolian to paroxysmal to, rarely, subPlinian. The range in activity exhibited by these volcanoes, from persistent to intermittent, and from effusive to explosive, provides a volcanological context for examining preserved melt compositions.

### (a) Stromboli and Etna, Italy

**Figure 7.**
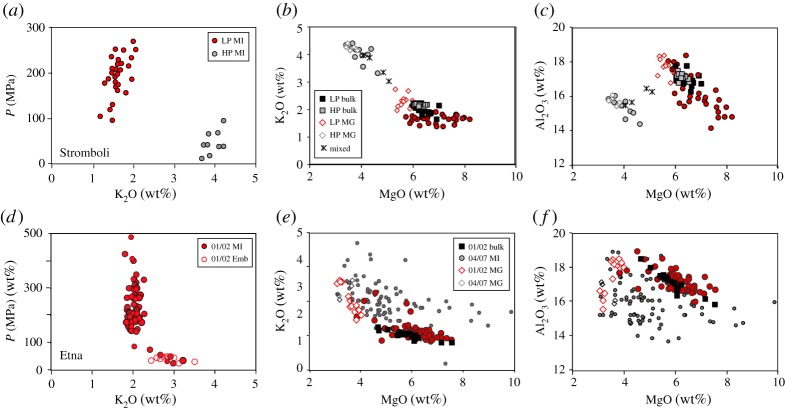
Melt compositions from Stromboli volcano, Italy (*a–c*) and Etna volcano, Italy (*d–f*). Stromboli shows LP data from paroxysmal eruptions (red) and HP data from normal Strombolian eruptions (grey). Bulk-rock data shown as squares (black for LP and grey for HP), melt inclusions as circles and matrix glass as open diamonds, except for mixed/mingled glasses, which are shown as (*). (*a*) Pressure versus K_2_O; (*b*) MgO versus K_2_O; (*c*) MgO versus Al_2_O_3_. Data from [31,95,99–101,103,104]. The same symbol shapes are used for Etna data, with red symbols for 2001–2002 data (black for bulk rock) and grey symbols for low-level activity of 2004–2007. (*d*) Pressure versus K_2_O; (*e*) MgO versus K_2_O; (*f*) MgO versus Al_2_O_3_. Data from [105–107].

Stromboli volcano is well instrumented and has a long history of petrologic studies, including extensive analysis and interpretation of quenched melt compositions (e.g. [95,99–102]). These data show that the eponymous activity involves frequent low-energy explosions that emit crystal-rich (HP or high-porphyricity) magma from storage regions located at less than 100 MPa (approx. 3 km below the summit). ‘Normal’ Strombolian activity is punctuated by occasional paroxysmal eruptions that erupt crystal-poor (LP or low-porphyricity) magma, with olivine-hosted melt inclusions that record magma storage at temperatures of 1140–1200°C, minimum pressures of 150–280 MPa (figure 7*a*), and periodic recharge of the deep storage system with CO_2_-rich basalt. Importantly, separate HP and LP magma storage regions have been maintained over time, with the deep magma erupted only during paroxysmal activity. Suggested triggers for paroxysms include (1) rapid rise of volatile-rich magma [95,103], (2) collapse of a CO_2_-rich bubble layer [108] or (3) depressurization of the magmatic system during episodes of lava effusion (e.g. [96,109]).

The melt compositional characteristics of the HP (shallow Strombolian) and LP (deep paroxysmal) storage regions have been characterized [95,100,101,103]. Although the two magma types have similar bulk compositions (figure 7*b,c*), contrasting storage conditions are evident in both melt inclusion and matrix glass compositions. LP melt inclusions record crystallization between 300 and 100 MPa (minimum pressures; figure 7*a*) of olivine and, primarily, clinopyroxene [95]; the trend of increasing Al_2_O_3_ with decreasing MgO shows that plagioclase was not part of the crystallizing assemblage. Additionally, extension of melt inclusion compositions to higher MgO than the bulk compositions indicates initial crystallization from a more Mg-rich melt. The total amount of crystallization is modest, however, as indicated by the limited variation in incompatible element K_2_O. LP matrix glass compositions sit at the evolved end of the melt inclusion trend; Al_2_O_3_^max^ suggests H_2_O_plag-in_ at approximately 3.6 wt% (figure 4*a*), which is within error of the maximum measured H_2_O contents in Stromboli melt inclusions (3.8 wt% [95]). High-Al_2_O_3_ matrix glass compositions suggest that the upper part of the LP magma storage region is close to H_2_O-saturated. Olivine-hosted melt inclusions in HP magmas, in contrast, have lower MgO and Al_2_O_3_, and higher K_2_O, consistent with extensive shallow crystallization of plagioclase [95,110]. The host olivines are also more evolved than those in the LP magma. Matrix glass compositions sit at the evolved end of the HP melt inclusion trend, with K_2_O contents that require an additional approximately 50% crystallization of the average LP matrix glass. Critically, intermediate melt compositions are absent, except when produced by mingling of HP and LP magma during paroxysmal eruptions (figure 7*b,c*). The lack of intermediate compositions requires isolation of the two magma storage regions, as well as rapid magma transfer from deep storage regions to the surface during paroxysmal activity [95].

Etna volcano is characterized by eruptive activity that is more varied in style and frequency than Stromboli, ranging from effusive to Strombolian, violent Strombolian and, in the past, Plinian. Although primary magma compositions have varied in time, including during recent activity, there are also numerous examples of the same bulk composition recording different trajectories in *P–T*–*φ* space, often in association with changes in eruptive style. Here, we compare melt inclusion and matrix glass data from highly explosive eruptive episodes in 2001–2002 [105,106] with similar data from effusive and low-level explosive eruptions in 2004, 2006 and 2007 [107]. The former included violent Strombolian eruptions from different vents; we use data from only the lower 2001 vents, which record the introduction of new magma into the system [105], as well as data from activity in October to November 2002 [106]. The latter were degassed and comprised mostly lava flows [107]; elevated measurement of CO_2_/SO_2_ ratios prior to individual effusion events, however, suggests that these flows were fed by ascent of different magma batches from depth [111,112].

Bulk-rock compositions from 2001 to 2002 are somewhat more variable that those of Stromboli LP magma, although estimated magma temperatures of 1125–1160°C are more restricted [106]. Olivine-hosted melt inclusions from these highly explosive eruptions track crystallization of mafic phases over a minimum pressure range of 500 MPa to near-surface values (figure 7*d*). Variations in K_2_O are limited except in the few low-pressure melt inclusions (less than 100 MPa) from eruptive activity in late 2002 (figure 7*d,e*). The maximum Al_2_O_3_ values are found at MgO of approximately 4.5 wt%, concurrent with the most evolved higher-pressure MI compositions (figure 7*f*). Application of the Al_2_O_3_ hygrometer (figure 4*a*) suggests H_2_O_plag-in_ less than or equal to 4 wt%, again within error of the maximum measured H_2_O in melt inclusions. Variations in matrix glass compositions require approximately up to 40% syn-eruptive decompression-driven crystallization, consistent with observed moderate to highly crystalline groundmass textures. In summary, melt compositions from the 2001 to 2002 activity, like the volatile data, provide evidence of magma extraction from a storage region that spanned a substantial *P* range, and extracted bulk magma compositions that define a near-continuous LLD trend with the melt inclusion and matrix compositions. This continuous trend contrasts with the compositional gaps in melt compositions observed at Stromboli, suggesting that a more vertically continuous magmatic system was required to maintain the sustained activity observed during the 2001–2002 eruptive episode at Etna.

Melt inclusion data from the mostly effusive 2004–2007 samples, in contrast, form a diffuse trend in K_2_O–MgO space that is parallel to, but elevated above, the 2001–2002 data and also extends to higher MgO (figure 7*e,f*, grey symbols). Although high K_2_O could reflect extensive PEC, this does not explain the high-MgO end of the trend, which instead suggests at least limited input of more primitive magma [112]. The host olivine compositions overlap with the olivine hosts of the 2001–2002 sequence, but extend to substantially lower forsterite contents [106]. The melt inclusion data also have highly variable Al_2_O_3_, although they reach an Al_2_O_3_^max^ that is similar to that of the earlier explosive sequence. Matrix glass compositions are similar to those of 2001–2002 and less evolved than some melt inclusions, which requires entrainment of crystals from shallower, cooler and/or more H_2_O-poor regions of the magmatic system.

Melt inclusion volatile contents are low in H_2_O but maintain substantial CO_2_ [107]; these data led Collins *et al*. [107] to suggest that the melt had equilibrated with fluxing CO_2_-rich fluids prior to eruption, consistent with the observations of CO_2_-rich gas emissions that preceded effusive events [111]. A potential problem with this interpretation is the nature of the samples, which include (slow-cooling) lava, where MIs are prone to diffusive H_2_O loss [40]. That said, the glass data require a range of storage conditions for the late-erupted lavas, as well as extensive plagioclase crystallization that is consistent with, but does not require, gas fluxing (or ‘flushing’). Arguments for the importance of CO_2_ flushing at Etna are also provided by Caricchi *et al*. [113].

### (b) LLaima, Chile

Llaima volcano is located in the Southern Volcanic Zone of Chile. Although not persistently active, the volcano has had several historic eruptive episodes, most recently in 1955–1957 and 2008–2009. Detailed studies of the eruptive products of these violent Strombolian paroxysms, as well as those of fissure-fed eruptions in 1850, suggest that, in contrast to Etna and Stromboli, the magmatic system beneath Llaima comprises a relatively shallow complex network of dykes/sills that feeds individual eruptions; the extent to which individual melt bodies are isolated except during periods of eruptive activity is a topic of discussion [114–117]. The dominant crystal phases are plagioclase and olivine. Zoning in both phases preserves evidence of frequent recharge by volatile-rich mafic magma; the recharge and resident magmas are ‘broadly co-genetic’ and linked via variations in the extent of olivine crystallization [114,115].

**Figure 8.**
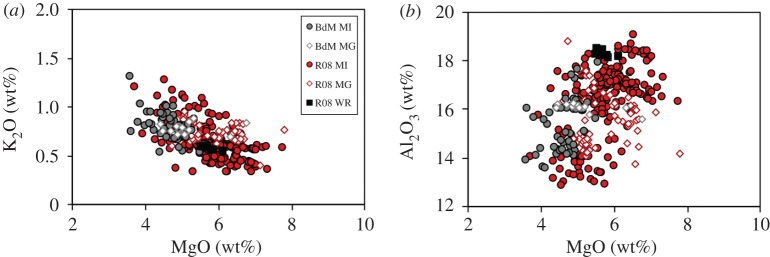
Melt compositions from Llaima volcano, Chile (BdM from [114]; R08 from [116]). Black squares show bulk composition, circles show melt inclusion data from the 2008 eruptive activity, open diamonds are matrix glass. (*a*) MgO versus K_2_O; (*b*) MgO versus Al_2_O_3_.

The most extensive documentation of melt compositions exists for the most recent eruption of Llaima (2008–2009) [114–117]. The bulk composition of the erupted samples shows only a limited range, which lies within the compositional range of olivine-hosted melt inclusions (figure 8). Melt inclusion compositions are similar to those of Stromboli and Etna, although they extend to both lower K_2_O and Al_2_O_3_ values, and the crude positive correlation of Al_2_O_3_ and MgO records the co-crystallization of olivine and plagioclase phenocrysts, the main phenocryst phases. Melt inclusion trace element measurements show typical arc signatures with moderate enrichment of light rare-earth elements, and overlap with bulk compositions [116]. In detail, they show a negative correlation of Sr/Zr and Al_2_O_3_, which can be reproduced by models with less than or equal to 55% crystallization, or close to the nominal ‘eruptible’ limit [114]. We note, however, that the compositional variation shown in figure 8 is difficult to reconcile with a simple LLD, given the variation in both K_2_O and Al_2_O_3_ at a single MgO, and the associated lack of apparent pressure dependence of the Sr/Zr ratio [114], which makes it difficult to place magma evolution within *P–T* space.

Further insight is provided by the matrix glass compositions, which are unusual in that they lie *within* the much larger compositional range of the melt inclusions, rather than at the evolved end of this range. Similarly, the average matrix glass temperature (1140°C; range 1114–1183°C) is higher than the average melt inclusion glass temperature (1118°C; range 1013–1198°C) [118], and olivine compositions are both more and less evolved than expected for equilibrium with associated melt inclusions. Evolved melt inclusion compositions may reflect either PEC or crystal entrainment from more solidified portions of the magmatic system (e.g. [97]). The latter interpretation is supported by the same trend in PEC-corrected melt inclusions and the numerous glomerocrysts observed within the 2008 scoria, which are inferred to be samples of erupted crystal mush [114,116]. There is also evidence of both assimilation (low TiO_2_, K_2_O and high Al_2_O_3_) and fractionation at a high modal abundance of plagioclase (high TiO_2_, P_2_O_5_, K_2_O and low Al_2_O_3_, CaO). Taken together, the preserved melt compositions in Llaima samples support interpretations [114,116,117] of relatively shallow magma storage, rapid syn-eruptive evacuation of the volcanic conduit, and entrainment of crystals from parts of the system that have experienced different amounts of fractionation and assimilation.

### (c) Fuego, Guatemala

Fuego volcano erupts frequently in styles that range from Strombolian to paroxysmal to, on rare occasions, subPlinian [37,119]. Strombolian eruptive products are difficult, if not impossible, to sample directly because of their limited dispersion outside of the vent region. Paroxysmal eruptions, in contrast, vary in size and energy, with the largest generating substantial pyroclastic density currents (PDCs) in addition to widespread ashfall (e.g. 3 and 5 June 2018). The most recent very large (subPlinian) eruption occurred in 1974; it lasted from 14 to 23 October and comprised several explosive events, the largest of which was on 17 October. All eruptive phases were well documented and many were sampled [37]. Here, we examine the compositional data of glass phases from both the 1974 subPlinian eruptions and later paroxysmal eruptions; to the published data we add matrix glass analyses from one 1974 ash sample, two clasts from 1974 PDCs, and three PDC clasts from a paroxysmal eruption in 2012 (see the electronic supplementary material for sample descriptions, analysis methods and data table).

**Figure 9.**
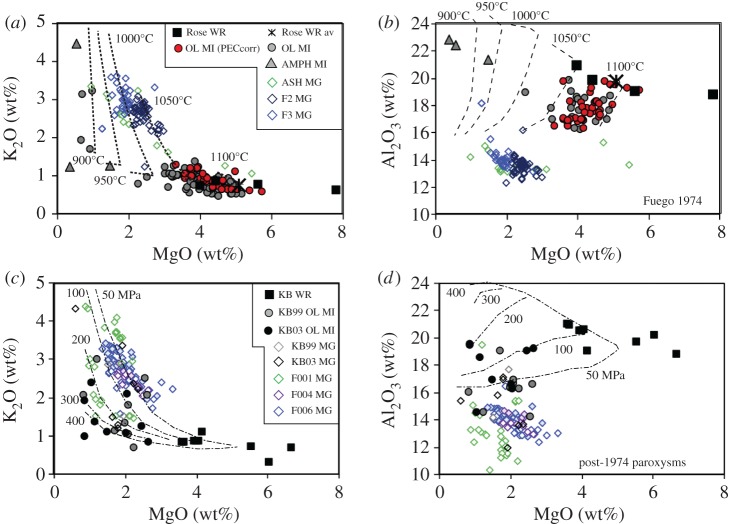
Melt compositions from Fuego volcano, Guatemala. Data are plotted separately for the 1974 subPlinian eruptions (*a,b*) and the sub-1974 paroxyms (*c,d*). (*a,c*) MgO versus K_2_O; (*b,d*) MgO versus Al_2_O_3_. Data from [36,120–122]. Also marked are the trends from figure 3 for ITD at a range of temperatures (as labelled). (*a,b*) Whole-rock data from Rose ([37]; WR); olivine-melt inclusions (OL MI, from [36,120,121]); red circles represent the largest explosion on 17 October 1974 and have been corrected for PEC (from [40]); grey triangles are amphibole melt inclusions (AMPH MI) from [36]; ash matrix glass (MG) from samples described in electronic supplementary material. (*c*,*d*) Whole-rock data (KB WR) from [36]; melt inclusion data plotted separately for paroxysms from 1999 (KB99) and 2003 (KB03; both from [36]); diamonds are matrix glass data from 1999 and 2003 (KB99, KB03) and from 2012 (from samples described in the electronic supplementary material).

The products of the 1974 eruption have been the subject of numerous studies, starting with a detailed analysis of both the physical and compositional characteristics of deposits from the four main eruptive phases (10–24 October [35]). Important observations from this study include variations in the abundance of olivine and plagioclase through time, with corresponding variations in the bulk compositions of ash samples (figure 9), as well as a wide compositional range of melt inclusions in olivine (K_2_O = 0.57–1.32 wt%), plagioclase (K_2_O = 0.5–1.58 wt%) and magnetite (K_2_O = 0.84–1.01 wt%). Calculated olivine-melt temperatures of the erupted magma ranged from 1010°C to 1130°C, and an inverse relation between S and K_2_O suggested crystallization over a pressure range. Finally, Anderson [35] demonstrated that, although the eruption tapped material from a range of depths and temperatures, the overall trend was of that of tapping an increasingly deeper source through time. Subsequent petrologic studies have focused primarily on olivine-hosted melt inclusions (e.g. [36,40,120,121]) and have confirmed the variability of both melt inclusion and matrix glass compositions in addition to showing that the volatile contents (H_2_O ≤ 6.5 wt% and CO_2_ ≤ 4500 ppm [120,121]) require pre-eruptive storage in a magma reservoir that probably extended to the mid-crust (greater than 500 MPa [123]). The temperatures of melt inclusion entrapment in olivine [36] overlap early estimates (1020–1100°C); rare amphibole inclusions in approximately An_91_ plagioclase megacrysts, however, suggest lower equilibration temperatures (approx. 988 ± 22°C using the formulation of [36,124]) and confirm early crystallization at high pressures (greater than 500 MPa).

In detail, olivine-hosted melt inclusions from 1974 samples show a compositional range dominated by variations in MgO, but with increases in K_2_O that require approximately 50% crystallization (figure 9*a*). Al_2_O_3_ is variable, but generally decreases with decreasing MgO (figure 9*b*), consistent with crystallization of olivine, clinopyroxene and plagioclase over a range of pressure and temperature (e.g. [40]). Rare amphibole-hosted MIs [36] show strong depletions in MgO and elevated Al_2_O_3_, suggesting extensive crystallization of mafic phases prior to feldspar saturation. Although Al_2_O_3_^max^ values lie outside the calibration range of the hygrometer (figure 4*a*), a linear extrapolation of the calibration suggests H_2_O_plag-in_ = 7.6–9.6 wt%. These values are similar to H_2_O contents of high-Al basalts from the Lesser Antilles, which are also characterized by deep-crystallizing amphibole [125]. Amphibole crystallization prior to plagioclase crystallization is also consistent with the low K_2_O of two melt inclusions, although in this context the high reported K_2_O of one inclusion is puzzling. Predictably, matrix glass compositions record decompression-driven crystallization of plagioclase and pyroxene (the dominant groundmass phases) that form a broad LLD trend in MgO–K_2_O space. Close examination, however, shows differences among samples. The ash sample (red open diamonds) preserves a nearly continuous compositional range from the most evolved olivine-hosted melt inclusions to a highly evolved end member, while matrix glass data from PDC samples (black and grey open diamonds) are more restricted in range and generally higher in MgO for the same K_2_O. Matrix glass data thus confirm extensive late-stage plagioclase crystallization, possibly over slightly different decompression paths.

Data from post-1974 paroxysmal eruptions, in contrast, show a range in bulk compositions, most probably the result of variable abundance of glomerocrysts, which are common in these samples [36]. Olivine-hosted melt inclusions have K_2_O contents that vary by a factor of six, indicating highly variable crystallization histories (figure 9*c*). Al_2_O_3_^max^ is comparable to that of olivine-hosted MIs from 1974 (approx. 19.8, or H_2_O_plag-in_ ≈ 5.5 wt%), but is achieved at lower MgO (figure 9*d*). Glass compositions of both the matrix and interstitial melt from glomerocrysts track a broad decompression-driven crystallization path, although the inter-sample variability, particularly at low MgO, suggests that individual samples may preserve magma aliquots that have ascended via different d*P*/d*T* paths. More curious is the apparent trend in the matrix glass compositions of increasing Al_2_O_3_ with decreasing MgO, which is not anticipated given the high plagioclase content of Fuego pyroclasts.

The data shown in figure 9 can be compared with the model data shown in figure 3, with the important caveat that MELTS runs were designed to examine trends but not to reproduce, exactly, the Fuego system (for example, they do not include CO_2_ or other volatiles, nor do they include deep amphibole crystallization). For clarity, we show model trends for ITD in figure 9*a,b*, and model trends for IBC in figure 9*c,d*. Most of the 1974 MI data are bracketed by decompression–crystallization trends at approximately 1050–1100°C, consistent with geothermometry estimates. Matrix glass compositions follow the same trend, although at slightly lower temperatures (1000–1050°C). Exceptions include a few olivine-hosted MIs and the amphibole-hosted MIs, which suggest early IBC at high pressure prior to rapid decompression, as also indicated by thermobarometric evidence for early and deep amphibole crystallization [36]. Melt inclusion data from post-1974 paroxysms, in contrast, follow IBC trends on the plot of K_2_O versus MgO, while matrix glass compositions from two samples record decompression paths that are similar to 1974. A third sample (F001) preserves glass compositions that overlap with the olivine-hosted melt inclusion glass compositions of [36]. We note that the Al_2_O_3_–MgO trends are not well matched by our MELTS model, which probably reflects the open system behaviour of Fuego with respect to volatiles (which we did not attempt to model in MELTS). This mismatch notwithstanding, the data show that paroxysmal eruptions extract magma from a large *P–T* range, which is recorded in the melt compositions in addition to the phenocryst assemblage and volatile content of melt inclusions. Rapid magma extraction from a range of pressures and temperatures may also help to explain the apparent trend of increasing Al_2_O_3_ with decreasing MgO, which could represent magma aliquots that track parallel d*P*/d*T* trends.

In summary, melt inclusion data from mafic arc volcanoes record the *P–T* conditions in the magma storage region, while matrix glass data preserve information on magma ascent paths. All examples record pre-eruptive magma storage over a range of pressures and temperatures, and contribute to the view of arc magmatic systems as primarily vertically, rather than laterally, extensive. Olivine-hosted melt inclusion data differ, however, in the extent to which they record crystallization of mafic phases alone (explosive eruptions of Etna and Stromboli) or co-crystallization of mafic phases and plagioclase (Llaima and Fuego), which in turn records the depth of magma storage relative to the pressure of H_2_O saturation. Comparison of melt inclusion and matrix glass compositions could further be used to calculate the rate of syn-eruptive magma ascent, if the kinetics of decompression-induced crystallization were well constrained [30]. For example, individual paroxysmal eruptions of Stromboli typically last less than 60 s, and as a result the LP matrix glass shows little evidence of syn-eruptive crystallization (e.g. [126]). Explosive eruptions from Etna, in contrast, are more protracted, and matrix glass compositions record more of the decompression history. The magma storage location and resulting extent of (relatively shallow) plagioclase crystallization may also affect eruption style, as indicated by the difference in matrix glass compositions in explosive and effusive phases of recent Etna eruptions. Finally, the variability of melt inclusion and matrix glass compositions within samples requires that individual clasts, and individual crystals within those clasts, be assembled syn-eruptively from discrete parts of the magmatic system. The insight provided by melt compositions into the physical nature of both the magma storage region and the eruptive process is thus complementary to evidence provided by the crystal cargo and volatile contents of crystal-hosted melt inclusions.

## 5. Discussion and conclusion

The review of mafic melt compositions provided above illustrates the variability of melt compositional data, and the insight it provides into the structure and dynamics of TCMS. The overarching conclusion is that the products of individual eruptions commonly include melt (± crystals) drawn from an extensive pressure (and sometimes temperature) range. These different components may be intimately mixed, or rapidly mingled, during eruption. Critically, even when these melts are derived from the same starting composition, they cannot necessarily be related by simple LLD models, but instead may reflect rapid assembly from a range of *P–T* space, and corresponding range of d*P*/d*T* paths. Our review also highlights differences in conditions of magma storage and ascent between low-H_2_O (ocean island) and high-H_2_O (arc) magmas [93].

Melt compositions erupted from high-flux magmatic systems such as Kīlauea, Hawai‘i, record variations in initial melt inputs from mantle sources, can be used to reconstruct the volume of magma reservoirs, and reveal complex mixing and mingling of melts with a wide compositional range that reflect an equally large temperature range. What emerges is a picture of a shallow magma reservoir that is continually resupplied from depth, and which is underlain by a crystal-rich reservoir of primitive melt (and olivine) that is tapped only during unusually explosive eruptions, or by flank eruptions where magma has circumvented the shallow summit reservoir. Low-flux ocean island systems, in contrast, show little evidence for shallow magma storage but instead erupt magma that is sourced at, or below, the Moho. This is true of both alkalic (pre- and post-shield) stages of Hawaiian volcanism and in low-flux hotspot environments such as the Canaries.

Mafic arc magmas are distinct from ocean island magmas in their high H_2_O contents; as a result, melt compositions are strongly affected by d*P*/d*T* path. Similarities with Kīlauea include the common association of high-intensity eruptions with magma sourced from a wide pressure range (vertical extent) and the rapid ascent of deep-sourced magma, which seems most easily explained by a downward-propagating decompression wave (e.g. [96,109]). Also similar are accumulations of shallow-sourced magma in frequently active volcanic systems, where melt compositions are buffered either at a peritectic composition (e.g. for differentiated Kīlauea magmas [127]) or by mafic recharge (including volatiles; e.g. [113]).

A final question relates to the stability of different melt–mush configurations and their relation to local fluxes of magma and volatiles. Ocean island volcanoes show a direct relation between magma flux and depth of melt accumulation, with low-flux systems sourced from at or beneath the Moho; the same may be true for mid-ocean ridge systems [128]. Melts erupted from arc volcanoes, in contrast, are commonly sourced from mid- to upper-crustal levels. The extent to which mid-crustal magma accumulation regions are connected permanently, or only transiently, to shallower storage regions is not known. Stromboli presents an interesting example, in that it appears to be a long-lived system that maintains discrete deep and shallow magma reservoirs, which communicate only during paroxysmal eruptions.

In conclusion, we demonstrate that melt compositions provide a comprehensive record of magma evolution, accumulation, decompression and mixing that cannot be explained by simple LLD trends. Instead, observed compositional variations contain important information about the physical nature of TCMS across a range of tectonic settings, as well as paths of magma transport to the surface during eruption. Importantly, these compositional variations are supported by both experimental data and thermodynamic models, which are used to simulate fractionation at a range of pressures, thermal conditions and volatile contents. Finally, we note that detailed analyses of rhyolitic melts used to assess the chemical and physical nature of melt bodies that supply very large eruptions are providing evidence for equally diverse melt sources (e.g. [4,118,129,130]), and further illustrate the importance of combining melt and crystal analyses to fully characterize magmatic systems.

## Supplementary Material

Sample description and analytical methods

## Supplementary Material

Table of analyses

## Data accessibility

The datasets supporting this article have been uploaded as part of the electronic supplementary material.
