# Supplementary material for: Mafic glass compositions: a record of magma storage conditions, mixing and ascent
Source: Philos Trans A Math Phys Eng Sci. 2019 Jan 7;377(2139):20180004. doi: 10.1098/rsta.2018.0004 (PMC6335480; doi:10.1098/rsta.2018.0004)
Supplement: Sample description and analytical methods [file rsta20180004supp1.pdf]

Supplementary Material - sample descriptions and methods

To supplement published data on (primarily) olivine-hosted melt inclusions from the 1974 and post-1974 eruptions of Fuego, we add microprobe measurements of groundmass glass. Samples were collected from pyroclastic density current deposits produced by the 1974 subPlinian eruptions (samples 1974 F2 and 1974 F3) and from a paroxysmal eruption in 2012 (2012 F1, 2012 F4 and 2012 F6). To these we added ash and lapilli samples from 1974 given to us by AS Lloyd (V36B, V36C). All samples are notable for the variability in groundmass textures (see thin section photos that follow), hence the large number of matrix glass analyses. Glass data include groundmass glass, glass preserved in crystal-hosted embayments, and glass preserved in glomerocrysts/small cognate nodules. Although these glass sources are identified separately, glass compositions overlap and therefore, for clarity, are not differentiated in Fig. 9.

Glass compositions were analysed using the JEOL 8530F Hyperprobe at the University of Bristol. Operating conditions include 20kV accelerating voltage, a 10 nA beam current, and a 10 μm beam size. Counting times were 10 seconds for Na, K, Si, Al, and Ca, 30 s for Ti and Fe, and 60s for Mg and S. Analyses are provided in the accompanying Excel spreadsheet, which provides analyses normalised to 100% plus original analysis totals.

1974 V36B

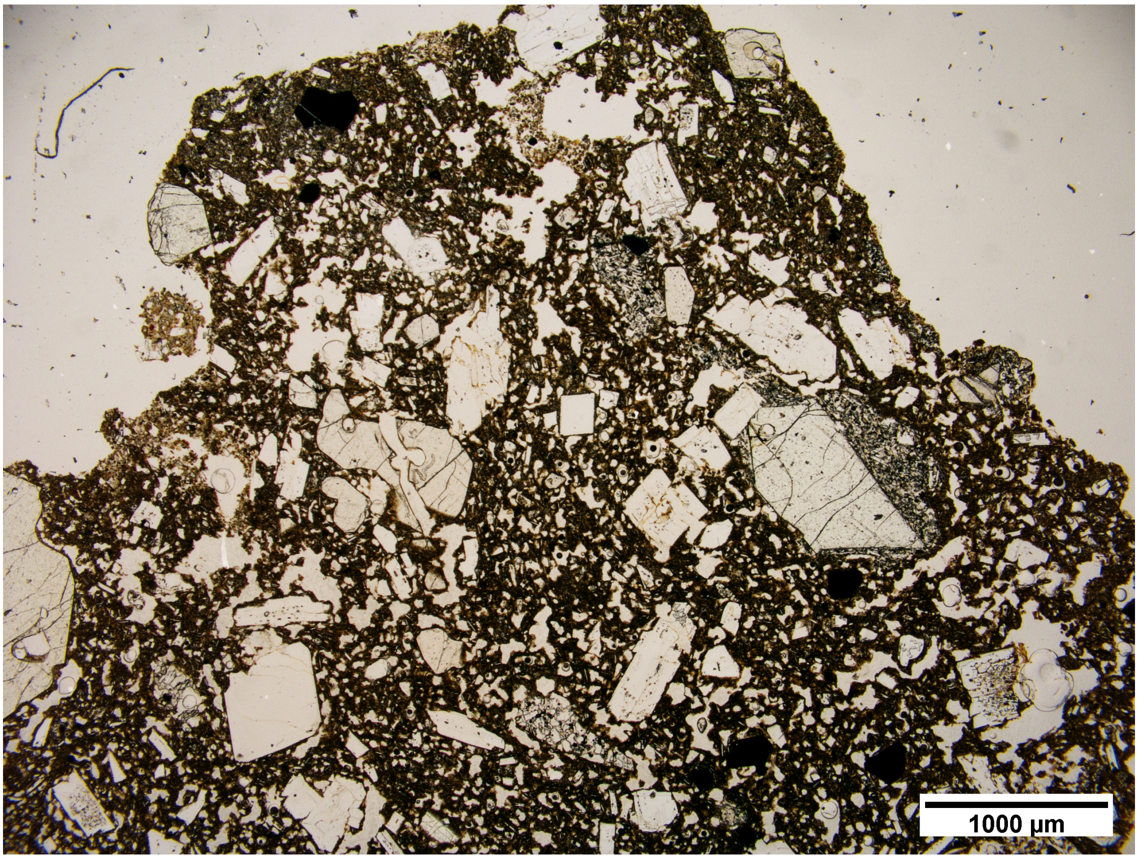

1974 V36C

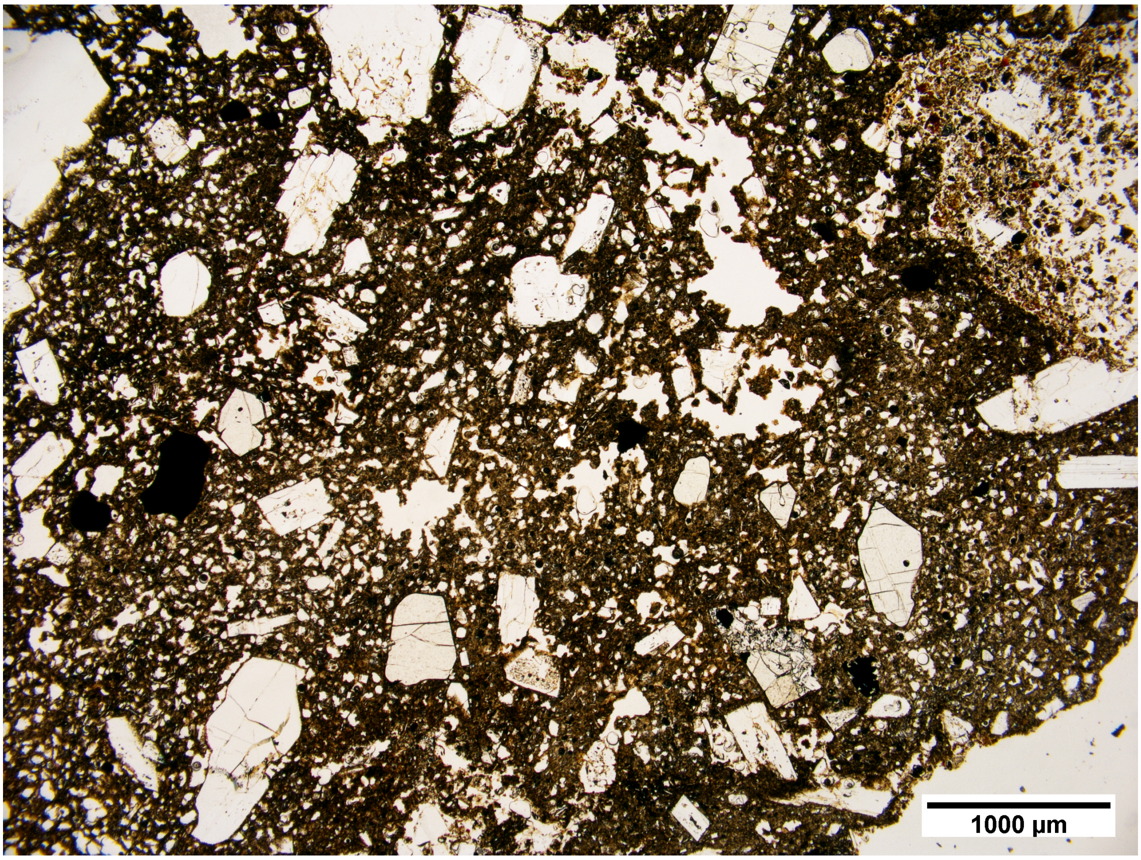

1974 F2a

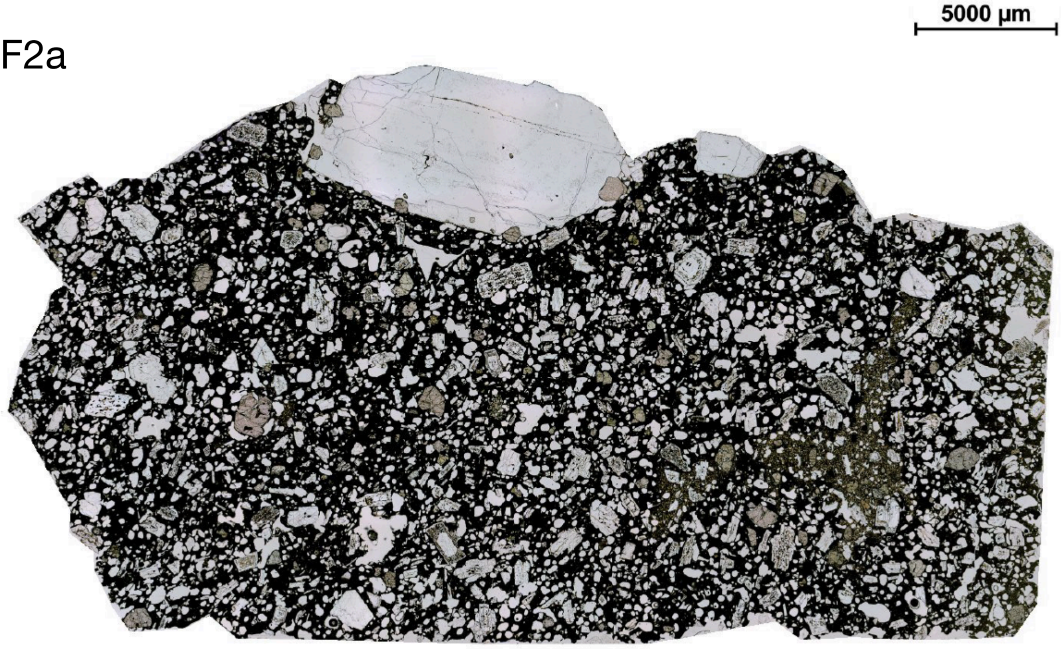

1974 F2b

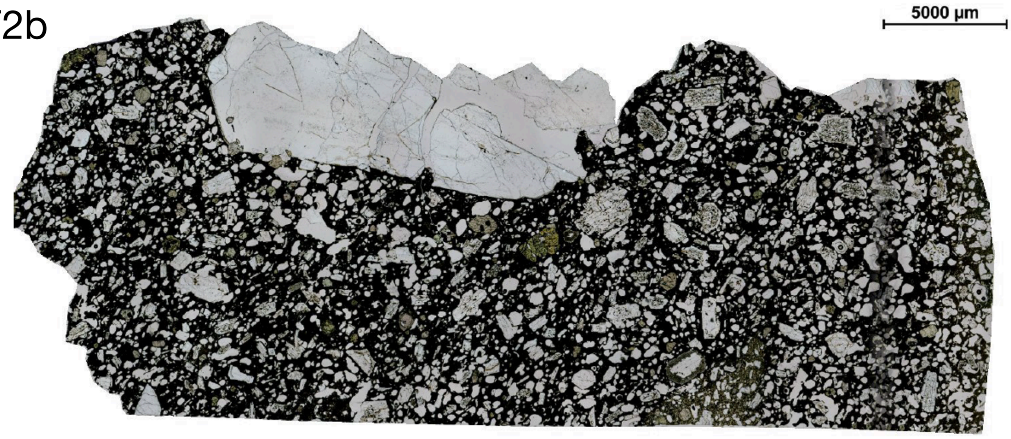

1974 F2c

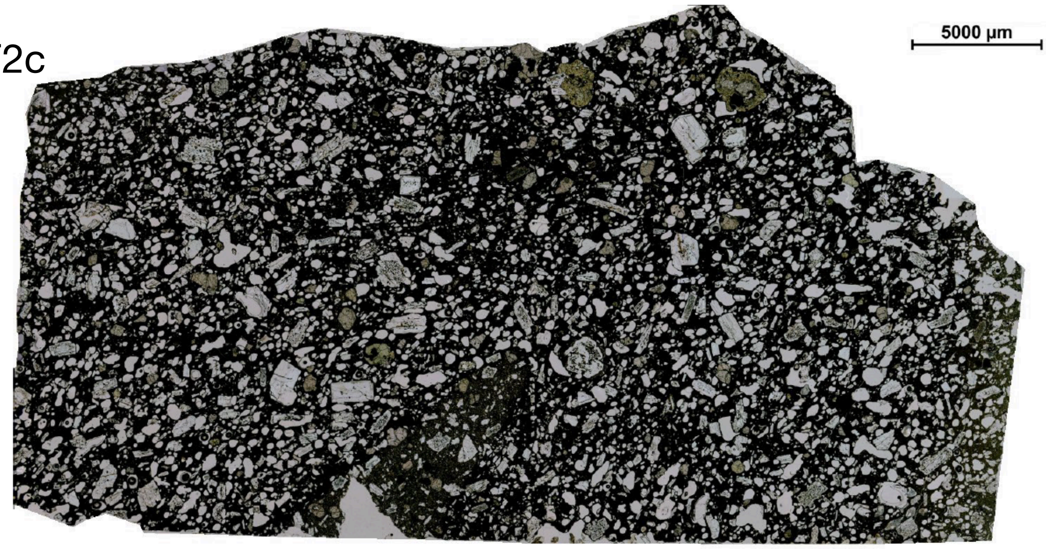

1974 F3

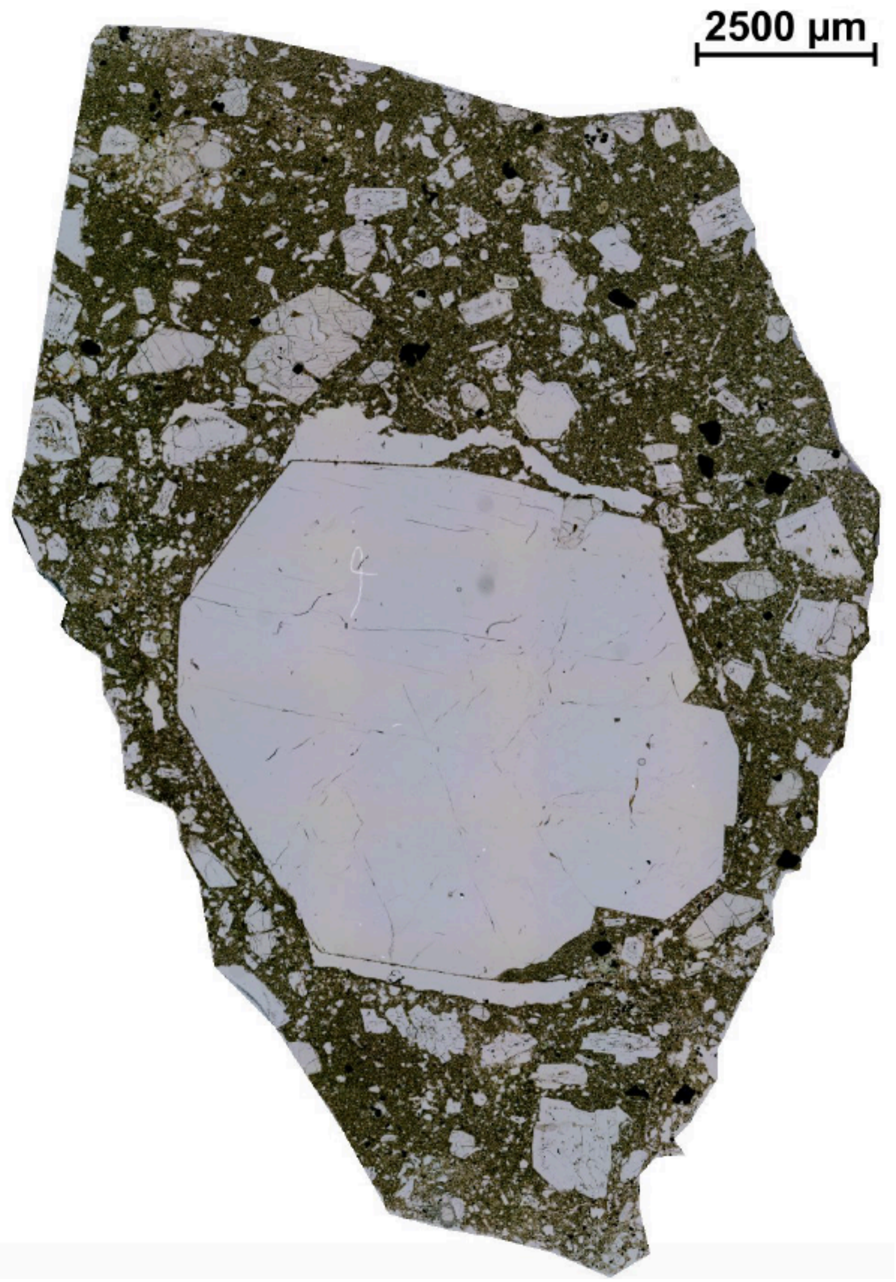

2012 F1

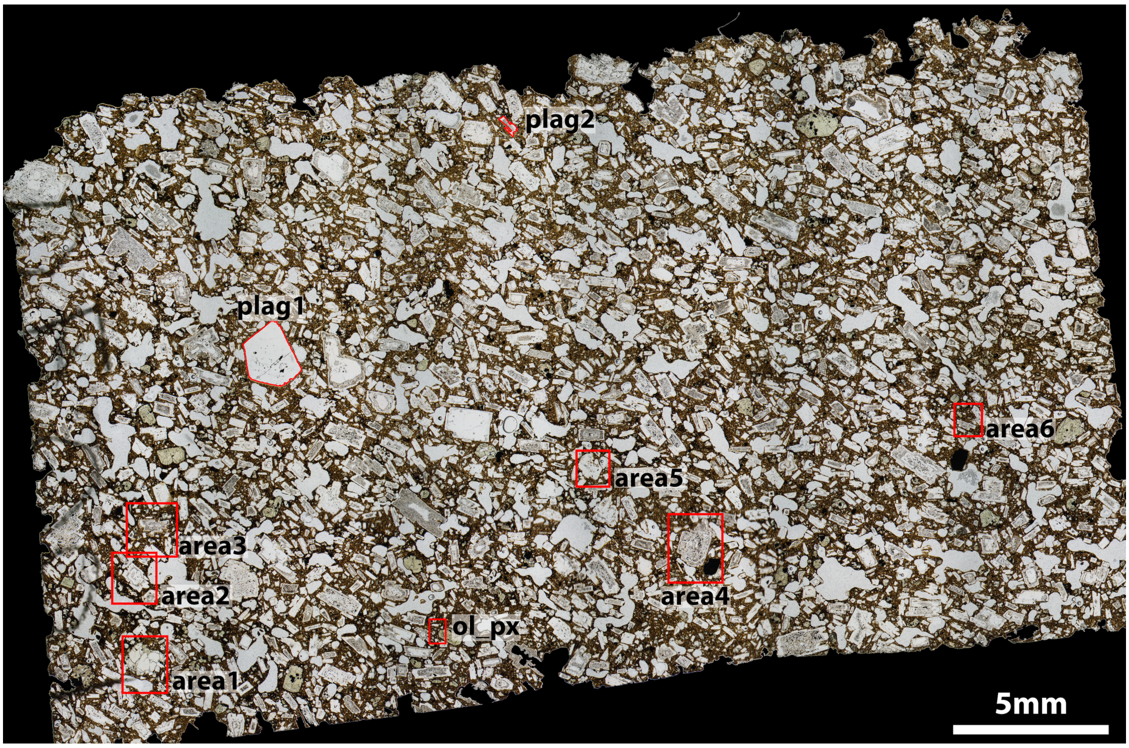

2012 F4

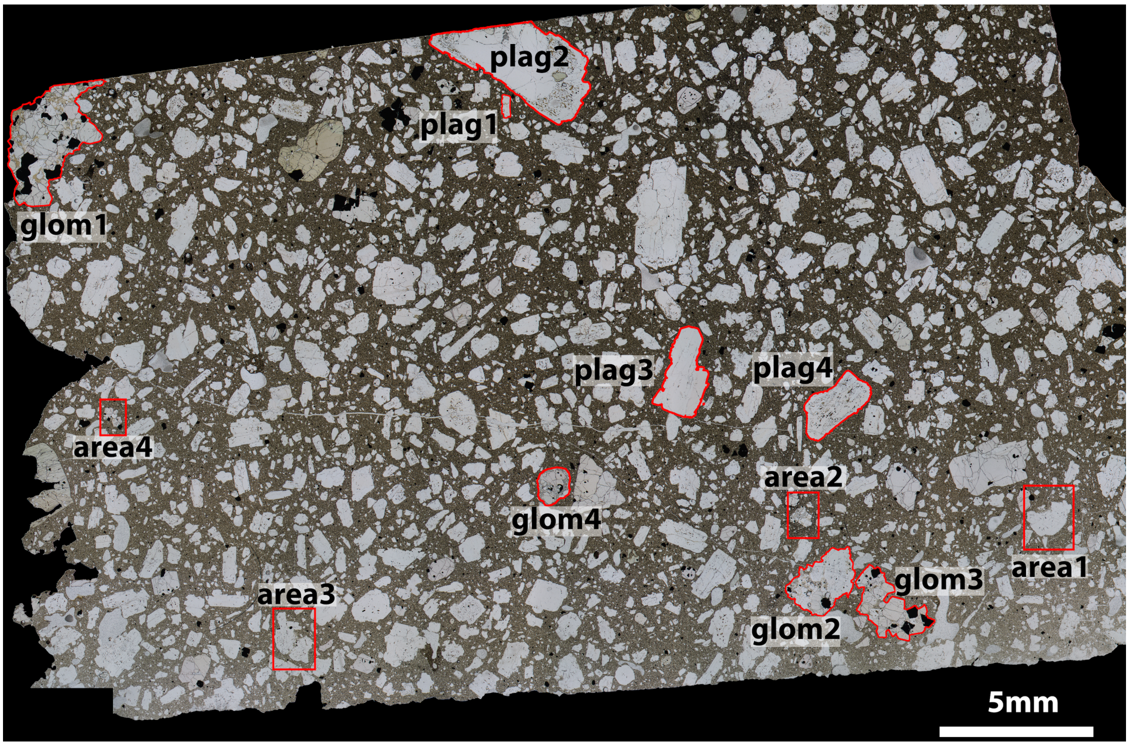

2012 F6

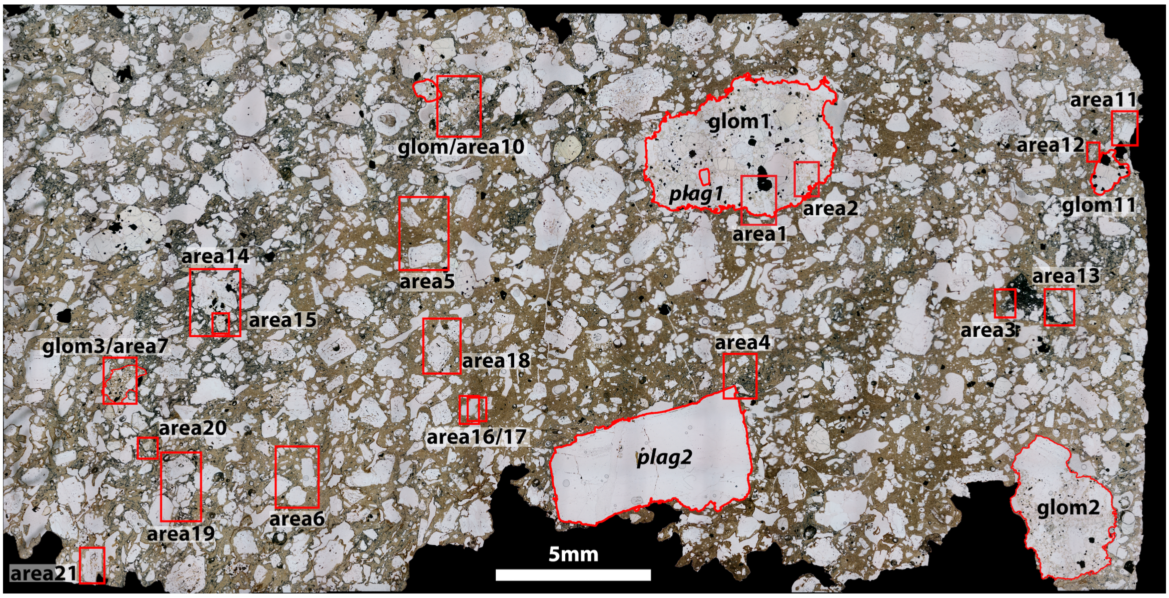
glom/area10
glom1
area11
area12
plag1
area2
glom11
area1
area14
area5
area13
area15
area3
glom3/area7
area18
area4
area20
area16/17
plag2
area6
area19
glom2
5mm
area21
